# Pyoderma gangrenosum-like sporotrichosis: case series of three patients and literature review^☆^

**DOI:** 10.1016/j.abd.2024.10.005

**Published:** 2025-05-06

**Authors:** Lucas Campos Garcia, Marianne de Sousa Nunes Soares, Gustavo Gomes Resende, Luciana Baptista Pereira

**Affiliations:** a Department of Dermatology, Faculty of Medicine, Universidade Federal de Minas Gerais, Belo Horizonte, MG, Brazil; b Department of Rheumatology, Universidade Federal de Minas Gerais, Belo Horizonte, MG, Brazil

Dear Editor,

Sporotrichosis is a subacute or chronic subcutaneous mycosis caused by thermodimorphic fungi of the genus *Sporothrix*.^1^ Although the diagnosis of the classic form is relatively straightfoward, other variants pose a significant diagnostic challenge. Morphologically, it can simulate keratoacanthoma, erysipelas, sarcoidosis and pyoderma gangrenosum (PG). The term pyoderma gangrenosum-like sporotrichosis (PGLS) is used to describe extensive ulcerative forms.^2^, ^3^, ^4^, ^5^, ^6^, ^7^, ^8^, ^9^, ^10^, ^11^ Three new cases are reported and previously published cases are reviewed.

A 48-year-old female patient presented with a painful ulcer on her right thigh for three months, with progressive enlargement and satellite lesions (Fig. 1). She had autoimmune hepatitis and had been treated with prednisone 1 mg/kg/day and azathioprine 3 mg/kg/day for five years. Tissue cultures were negative, and histopathology had shown granulomatous, suppurative panniculitis and vascular aggression. Given the hypothesis of PG, the patient received intravenous pulse therapy with methylprednisolone (1 g/day for three days) without improvement. As there was no response, a new biopsy was performed on the edge of the same lesion, one week after pulse therapy. Yeast-like structures stained with PAS (periodic-acid Schiff) were found in the subcutaneous tissue on histopathology (Fig. 2A). Simultaneously, patient’s pet cat was diagnosed with sporotrichosis through secretion culture (Fig. 2B). As the patient had hepatorenal syndrome, treatment with potassium iodide (2 g/day) was chosen despite being immunosuppressed. The patient showed almost complete healing within three months of treatment (Fig. 1), when she presented decompensation of the liver condition and died. Although potassium iodide-associated hepatotoxicity is rare, it may occur in patients with pre-existing liver disease. However, the Gastroenterology team correlated the outcome to the worsening of the underlying disease.

**Figure 1:**
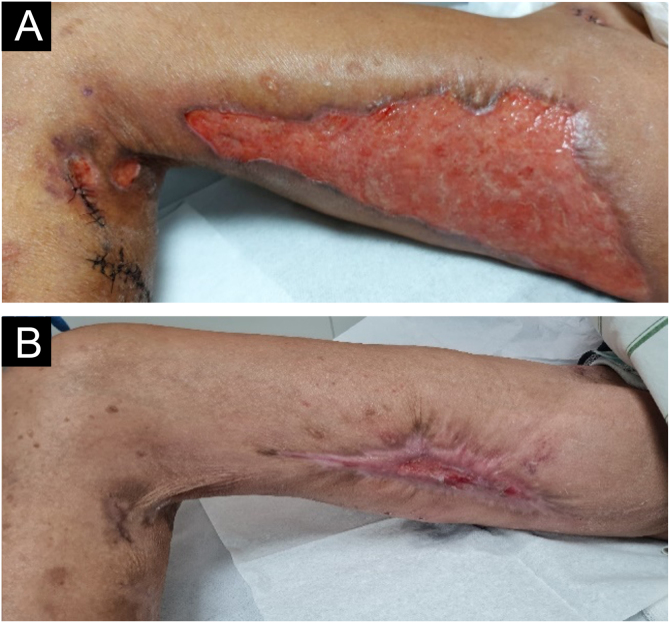
Pyoderma gangrenosum-like sporotrichosis. (A) Ulcer in the medial region of the right thigh with satellite lesions along the lymphatic tract. (B) Significant clinical improvement of the lesion after three months of treatment with potassium iodide.

**Figure 2:**
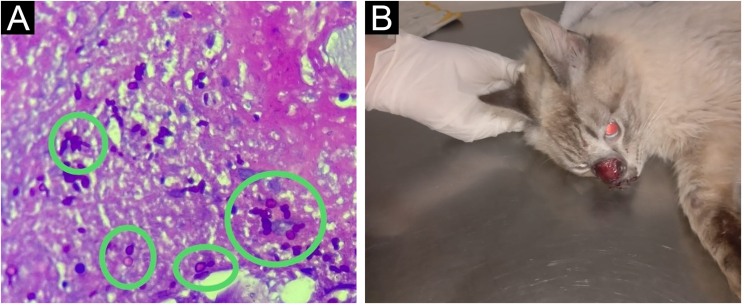
(A) Oval and elongated yeast-like structures in the deep dermis. (PAS, ×400). (B) Patient's cat with nasal ulceration diagnosed as sporotrichosis.

The second patient, a 49-year-old male, had a painful ulcer in the right antecubital fossa with satellite lesions and perilesional lymph node enlargement of one-month evolution (Fig. 3A). He had psoriatic arthritis and diabetes mellitus. He had been treated with infliximab 3 mg/kg for four years, sulfasalazine 2 g/day for three years, and prednisone 20 mg/day for six months. Based on the initial suspicion of ecthyma gangrenosum, clindamycin and ciprofloxacin were administered for one month, without improvement. Subsequently, *Sporothrix sp*. was isolated from the ulcer fragment and sent for culture. Histopathology revealed abscessed granulomas, but the fungal culture was negative. Itraconazole was started, but the patient developed hepatorenal syndrome, which led to drug discontinuation. As an alternative, potassium iodide (3 g/day) was administered for two months, with healing of the ulcer (Fig. 3B).

**Figure 3:**
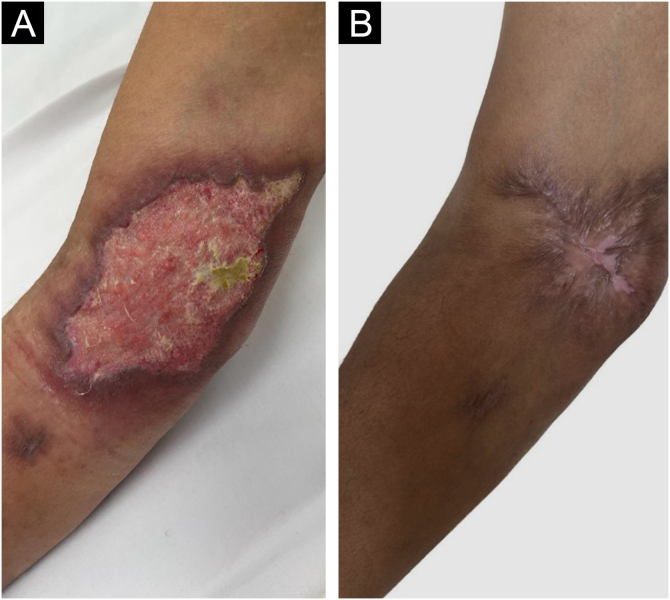
Pyoderma gangrenosum-like sporotrichosis. (A) Ulcer in the right antecubital fossa with satellite lesion. (B) Healing of the lesion after two months of treatment with potassium iodide.

The third patient, a 39-year-old male individual, presented a painful, ulcerated lesion with a cribriform appearance and satellite abscessed papules and nodules on the left leg of three-week evolution (Fig. 4A). Initial treatment with amoxicillin combined with clavulanate, clindamycin, vancomycin, and cefepime yielded no improvement. The patient had Crohn's disease and had received methotrexate 20 mg/week for four years and infliximab 5 mg/kg for two years. Given the initial hypothesis of PG, dapsone 100 mg/day was added for 30 days. Histopathology showed a neutrophilic inflammatory infiltrate and leukocytoclasia, abscessed granulomas and rare yeast-like spindle-shaped structures. In the culture, there was *Sporothrix sp.* growth, confirming the diagnosis. Itraconazole (200 mg/day) and potassium iodide (2 g/day) were started concomitantly, with healing after four months (Fig. 4B).

**Figure 4:**
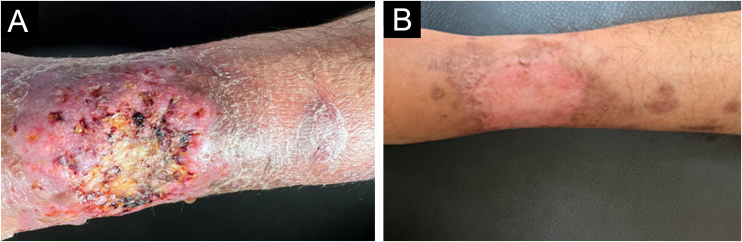
Pyoderma gangrenosum-like sporotrichosis. (A) Ulcer on the left leg with satellite lesions. (B) Healing of the lesion after four months of treatment with itraconazole and potassium iodide.

Extensive ulcerative forms of sporotrichosis are rarely described and occur especially in immunosuppressed patients. The term PGLS has been adopted due to the clinical and histopathological similarities with PG: phagedenic ulcers with erythematous edges, cribriform morphology and neutrophilic infiltrate.^3^ The initial approach to PG requires the exclusion of differential diagnoses through biopsy and culture. However, the search for pathogens is not always positive at first. The lack of response to immunosuppressants should motivate a diagnostic review. Furthermore, fungal structures are difficult to observe, making the diagnosis of sporotrichosis even more difficult.^3^ In this context, the culture of secretions or tissue fragments is extremely important,^11^ as well as a detailed anamnesis, especially regarding the presence of felines and risk activities, such as gardening.

Only 10 published cases of PGLS were found in the English-language literature in PUBMED (Table 1). More than half (n = 6) were published in the last six years, which may reflect an improvement in diagnostic accuracy or an increase in incidence, mainly due to the growing population of immunosuppressed patients. The cases reported herein were under immunosuppression.

**Table 1:** Cases published in the literature (PUBMED database, English language until July 2024).

	Gender/ Age	Immunosuppression/ Comorbidities	Lesion sites	Initial treatment	Diagnostic method	Treatment	Clinical outcome
Stroud et al. 1968^1^	M/62	Metastatic squamous cell carcinoma	R forearm	Await the investigation, despite the clinical suspicion of PG	+Tissue culture	Amphotericin B 25 mg/day for five days; potassium iodide added for 2 days	Death after seven days of treatment for AKI
Spiers et al. 1986^2^	M/46	No	Abdomen	Tetracycline, erythromycin, PDN, DDS, AZA	+Tissue culture	Potassium iodide 3 g/day	Clinical cure after two months
Wan-Qing et al. 1991^3^	F/56	Corticosteroid therapy for rheumatoid arthritis	L buttock and thigh	MDT for TB; PDN; DDS	+Secretion culture	Potassium iodide (6‒8 g/day)	Clinical improvement of the lesions. Death after two weeks due to pneumonia.
Byrd et al. 2001^4^	F/59	No	R leg	Systemic ATBs, PDN, AZA and cyclosporine	+Tissue culture; +PAS in AP	Itraconazole 600 mg/day, 18 months	Clinical cure after three months.
Lima et al. 2017^5^	F/39	No	Abdomen and R arm	PDN, immunosuppressors infliximab	+Tissue culture; +PAS yeast-like structures in AP	Liposomal amphotericin B (400 mg/day) six weeks + itraconazole 400 mg/day for 12 months	Clinical cure.
Charles et al. 2017^6^	F/57	Obesity and asthma	R arm	Levofloxacin, ceftriaxone, PDN, penicillin and topical clobetasol	+Tissue culture; +PAS yeast-like structures in AP	Itraconazole 400 mg/day	Clinical improvement and loss to follow-up
Takazawa et al. 2018^7^	M/47	Ulcerative colitis using mesalazine	R leg	Topical corticoids	+Tissue culture; +PAS yeast-like structures in AP	Potassium iodide 0.5 g for two weeks and 1 g for three weeks	Clinical cure in five weeks.
White et al. 2019^8^	M/62	Coronary artery disease	L thigh	Cephalexin, PDN, cyclosporine, ustekinumab, immunoglobulin	+Tissue culture and blood culture	Liposomal amphotericin B (5 mg/kg/day); itraconazole 600 mg/day; amphotericin (4 mg/kg/day); posaconazole 300 mg/day	Clinical cure after six months.
Saeed et al. 2019 ^9^	F/35	Alcohol abuse and type II diabetes	Legs, arms and abdomen	PDN, doxycycline	+Tissue culture; +PAS yeast-like structures in AP	Liposomal amphotericin B (5 mg/kg/day); posaconazole 300 mg/day; itraconazole 600 mg/day	Clinical cure after 12 months.
Tai et al. 2020 ^10^	M/78	Not reported	L arm	Cyclosporine, mycophenolate mofetil, PDN, ustekinumab and immunoglobulin	+Tissue culture	Itraconazole 200 mg/day for four months, developed liver and kidney failure, potassium iodide was started	Clinical cure after four months.
Case 1	F/48	Autoimmune hepatitis/use of PDN and AZA	R thigh	Systemic antibiotics and methylprednisolone	+PAS yeast-like structures in AP; +epidemiology	Potassium iodide 2 g/day for three months	Clinical improvement, death after three months due to hepatorenal syndrome.
Case 2	M/49	Psoriatic arthritis, atrial fibrillation, arterial hypertension, dyslipidemia, diabetes mellitus, use of infliximab, sulfasalazine, prednisone, amiodarone, warfarin	R antecubital fossa	Clindamycin and ciprofloxacin	+Tissue culture; Negative for yeast structures in PAS and Grocott	Itraconazole 200 mg/day, developed hepatorenal syndrome, started potassium iodide 3 g/day	Clinical cure after two months.
Case 3	M/39	Crohn's disease using methotrexate, infliximab and loperamide	L leg	Amoxicillin + clavulanate, clindamycin, vancomycin and cefepime, dapsone	+Tissue culture	Itraconazole 200 mg/day and potassium iodide 2 g/day	Clinical cure after four months.

PDN, Prednisone; AZA, Azathioprine; DDS, Dapsone; ATB, Antibiotics; PAS, Periodic Acid of Schiff; AP, Anatomopathological; MDT, Multi-drug therapy; TB, Tuberculosis; AKI, acute kidney insuficiency.

Interestingly, they had ulcers with a larger axis in the lymphatic drainage direction, while PG ulcers often have a more rounded shape. Satellite lesions occurred in the three cases and may help formulate the hypothesis of sporotrichosis.

Although potassium iodide is not the first therapeutic choice for immunosuppressed patients, it can be used as monotherapy when there are contraindications to other treatments.^3^, ^4^ In view of the experience in the first two cases, due to the synergistic drug effect and the report of the emergence of itraconazole-resistant *S. brasiliensis* isolates,^1^ the association was chosen for the third patient.

## Financial support

None declared.

## Authors' contributions

Lucas Campos Garcia: Study design, critical review, drafting and editing of the manuscript, review of the literature, approval of the final version of the manuscript.

Marianne de Sousa Nunes Soares: Drafting and editing of the manuscript, review of the literature.

Gustavo Gomes Resende: Drafting and editing of the manuscript, review of the literature.

Luciana Baptista Pereira: Study design, critical review, drafting and editing of the manuscript, review of the literature, approval of the final version of the manuscript.

## Conflicts of interest

None declared.
